# Cone-beam CT reconstruction for non-periodic organ motion using time-ordered chain graph model

**DOI:** 10.1186/s13014-017-0879-8

**Published:** 2017-09-04

**Authors:** Masahiro Nakano, Akihiro Haga, Jun’ichi Kotoku, Taiki Magome, Yoshitaka Masutani, Shouhei Hanaoka, Satoshi Kida, Keiichi Nakagawa

**Affiliations:** 1 Department of Radiology, The University of Tokyo Hospital, Bunkyo-ku, Tokyo, 113-8655 Japan; 2 Department of Radiation Oncology, The Cancer Institute Hospital, Japanese Foundation for Cancer Research, Koto-ku, Tokyo, 135-8550 Japan; 3 Faculty of Medical Technology, Teikyo University, Itabashi-ku, Tokyo, 173-8605 Japan; 4 Faculty of Health Sciences, Komazawa University, Setagaya-ku, Tokyo, 154-8525 Japan; 5 Faculty of Information Science, Hiroshima-City University, Hiroshima, 731-3194 Japan; 6 School of Medicine, Gunma University, Maebashi, 371-8511 Japan

**Keywords:** 4D imaging, Iterative reconstruction, Cone-beam CT, Chain graph model, Time-ordered organ motion

## Abstract

**Purpose:**

The purpose of this study is to introduce the new concept of a four-dimensional (4D) cone-beam computed tomography (CBCT) reconstruction approach for non-periodic organ motion in cooperation with the time-ordered chain graph model (TCGM) and to compare it with previously developed methods such as total variation-based compressed sensing (TVCS) and prior-image constrained compressed sensing (PICCS).

**Materials and Methods:**

Our proposed reconstruction is based on a model including the constraint originating from the images of neighboring time phases. Namely, the reconstructed time-series images depend on each other in this TCGM scheme, and the time-ordered images are concurrently reconstructed in the iterative reconstruction approach. In this study, iterative reconstruction with the TCGM was carried out with 90° projection ranges. The images reconstructed by the TCGM were compared with the images reconstructed by TVCS (200° projection ranges) and PICCS (90° projection ranges).

Two kinds of projection data sets–an elliptic-cylindrical digital phantom and two clinical patients’ data–were used. For the digital phantom, an air sphere was contained and virtually moved along the longitudinal axis by 3 cm/30 s and 3 cm/60 s; the temporal resolution was evaluated by measuring the penumbral width of the air sphere. The clinical feasibility of the non-periodic time-ordered 4D CBCT image reconstruction was examined with the patient data in the pelvic region.

**Results:**

In the evaluation of the digital-phantom reconstruction, the penumbral widths of the TCGM yielded the narrowest result; the results obtained by PICCS and TCGM using 90° projection ranges were 2.8% and 18.2% for 3 cm/30 s, and 5.0% and 23.1% for 3 cm/60 s narrower than that of TVCS using 200° projection ranges. This suggests that the TCGM has a better temporal resolution, whereas PICCS seems similar to TVCS. These reconstruction methods were also compared using patients’ projection data sets. Although all three reconstruction results showed motion related to rectal gas or stool, the result obtained by the TCGM was visibly clearer with less blurring.

**Conclusion:**

The TCGM is a feasible approach to visualize non-periodic organ motion. The digital-phantom results demonstrated that the proposed method provides 4D image series with a better temporal resolution compared to TVCS and PICCS. The clinical patients’ results also showed that the present method enables us to visualize motion related to rectal gas and flatus in the rectum.

## Introduction

The volumetric imaging technique using a cone-beam computed tomography (CBCT) device mounted on the gantry of a radiotherapy linear accelerator is crucial in both image-guided radiotherapy (IGRT) and adaptive radiotherapy (ART). Since first introduced by Jaffray et al. with a volumetric reconstruction algorithm widely known as the Feldkamp-Davis-Kress (FDK) method proposed by Feldkamp et al., [1, 2] it has enabled the highly accurate positioning of radiotherapy patients [3–5]. Recently, CBCT images have been expected to provide image sets as the material of a retrospective analysis for margin evaluation, anatomical deformation, and dose distribution [6–8]. Online four-dimensional (4D) CBCT reconstruction has already been used in the treatment of tumor sites with periodic motion, such as a lung tumor, and it has also provided an accurate setup that visually considers the intrafractional motion of tumors and risk organs [9–12]. Especially, in the scenario of adaptive radiotherapy, 4D CBCT and in-treatment CBCT imaging technique has been expected to enable understanding 4D dose distribution with better temporal resolution [13].

For the purpose of 4D CBCT image reconstruction accompanied by periodic organ motion such as respiration and cardiac activity, information related to the respiratory or cardiac motion signal is necessary to classify projections into several phase groups. In the case of respiratory motion, the measurement of a signal synchronized with projection images is categorized by two methods–the use of an external respiratory monitoring system and image-based respiratory phase recognition.

As an example of the latter case, Zijp et al. proposed the method known as the Amsterdam Shroud, which produces a 1D projection in the craniocaudal (CC) direction after applying a CC derivative filter,[14, 15] and this has been used in a commercial linac-integrated CBCT device, the X-ray Volumetric Imaging (XVI) System mounted on Elekta Synergy (Elekta, Crawley, UK). Regarding cardiac activity, Lauzier et al. demonstrated 4D image reconstruction using electrocardiogram (ECG) signals in order to sort projections into several cardiac phases [16].

In contrast to the various studies on volumetric 4D CBCT imaging with periodic motion, few studies have dealt with volumetric imaging with non-periodic intrafractional motion or deformation using CBCT in radiotherapy. In the modalities outside CBCT, volumetric imaging methods available for non-periodic motion monitoring have been introduced using MRI and transperineal ultrasound, but the former has required quite a huge system combined with MRI and linac and the latter has been limited to monitor organs around the anorectal area [17, 18]. Regarding CT and CBCT as an 4D volumetric imaging modality, the temporal resolution and continuous time-ordered image reconstruction have been intensely studied in the literature in the concept of short-scan CT image reconstruction [19–21]. In this approach, the temporal resolution is directly related to the angular range, and a shorter range surely provides better temporal resolution. However, it might cause a degradation in the image quality and artifacts if the range is shorter than 180° plus the fan angle [20]. On the other hand, Pang and Rowlands introduced “just-in-time tomography,” which reconstructs a digital tomosynthesis image from the projections of the cone-beam acquisition geometry [22]. The method was actually a time-ordered imaging approach, but the created tomosynthesis images were still two-dimensional.

To overcome the above limitations, iterative reconstruction approaches have been proposed and are still actively studied [16, 23–30]. In particular, compressed sensing (CS)-based iterative reconstruction approaches such as total-variation-based compressed sensing (TVCS) and prior-image constrained compressed sensing (PICCS) have enabled image reconstruction with a limited number of projections and have strengthened the reliability of 4D CBCT images [16, 23, 24, 31]. The key idea of CS-based image reconstruction method is that, the sparsified image can be reconstructed from an undersampled projection data set instead of directly reconstructing a target image [23]. The sparsified image, which is obtained by the application of sparsifying transform, contains significantly fewer image pixels which have significant pixel values. As the sparsifying transform, the *ℓ*_1_-norm of the local spatial gradient is widely used for image reconstruction, which is called the total variation (TV). TVCS employs the TV transform of the target image itself, and PICCS employs the one of the difference image between the target and the prior image [29].

The non-periodic-motion correlated image reconstruction approach is based on a method that bunches projections into several time-phase groups. In contrast to the periodic motion case, there is no need to measure the motion signals for phase binning. After classifying the projections with the constant projection-angle interval, the images can be independently reconstructed in each time-phase group. In principle, however, the reconstructed time-ordered image sets are correlated with the prior image, which is the image prepared in advance of the iterative process, and the image sets of previous or subsequent time phases in a series of continuous 4D images. In the present study, we explicitly regard the continuous 4D reconstructed image series as sequential data and express them in the manner of a chain graph model. This is formulated as the regularization term of the statistical image reconstruction [32]. In this study, we introduce a time-ordered 4D CBCT reconstruction method with the constraint of the time-ordered chain graph model (TCGM) and compare images reconstructed by previously introduced methods–TVCS and PICCS.

## Materials and Methods

**Fig. 1:**
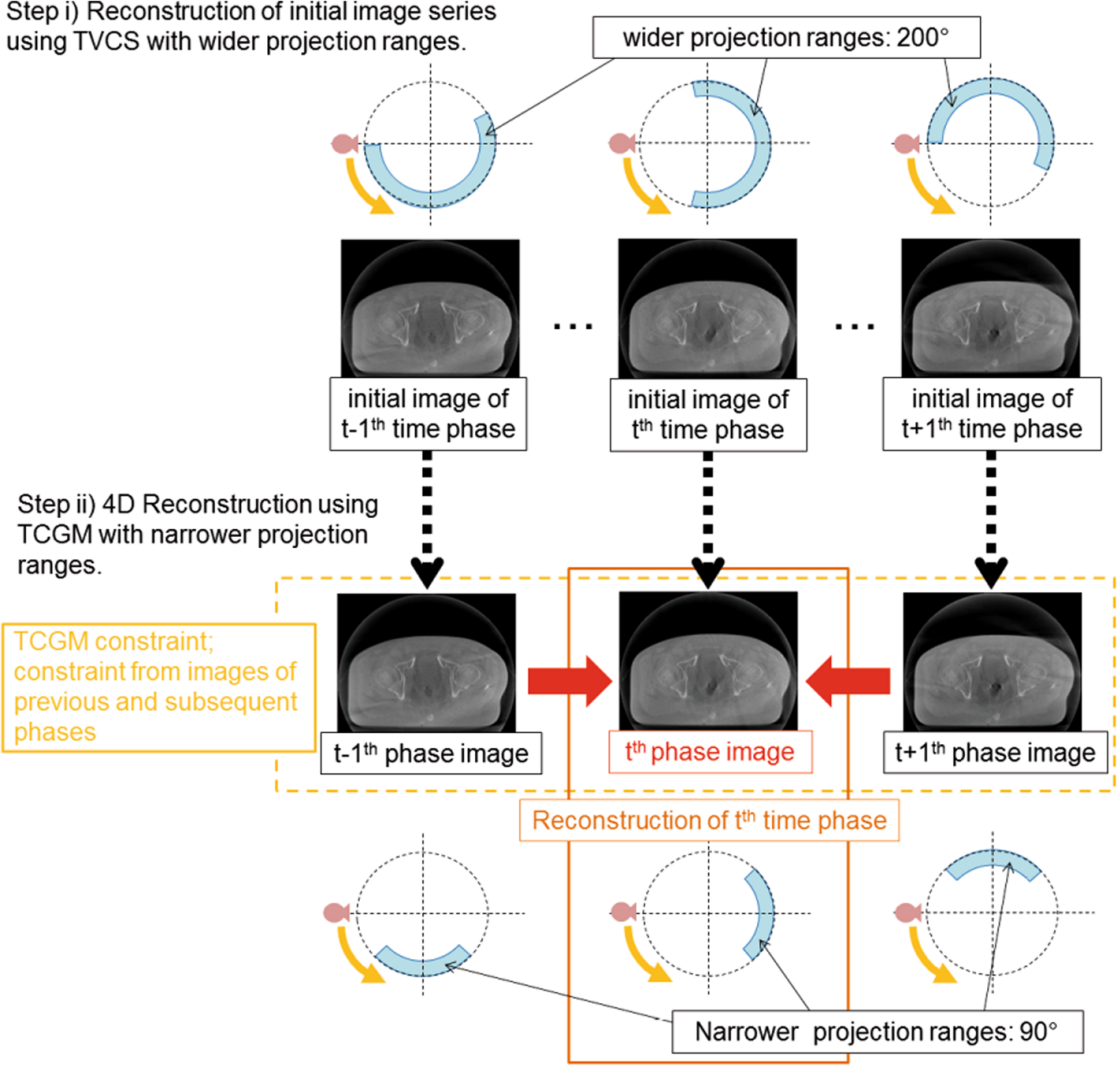
Image reconstruction workflow of the present method. The initial image series were reconstructed in Step i) by TVCS using wider angular ranges; then, PICCS or TCGM reconstruction was implemented in Step ii) using narrower angular ranges for each phase

In this section, the concept of the TCGM is described with the iterative reconstruction framework incorporating TVCS and PICCS. The reconstruction workflow is schematically summarized in Fig. 1, where the preprocessed time-ordered images are prepared with TVCS, and then, iterative reconstruction is carried out with a narrower projection angle. PICCS statically uses preprocessed images as a prior-image constraint, whereas the TCGM only uses them as initial data sets, and the constraint is dynamically imposed during the iteration step by the images reconstructed in the adjacent time phases. In the TCGM scheme, the reconstructed time-series images have a relationship with each other through a regularization term so that the time-ordered images are concurrently reconstructed.

The projection data sets used for image reconstruction will be also introduced in this section, which include those of a digital phantom with the virtual motion of an air sphere and those of two clinical patients’ data in the pelvic region.

### Brief review of statistical image reconstruction

In this study, the maximum *a posteriori* (MAP) approach was employed as our image reconstruction framework [25, 33–35]. The concept of MAP approach is to give a maximized probability of reconstruction images given projection images in contrast to the maximum likelihood expectation maximization (MLEM) approach, which gives a maximized probability of projection images given reconstructed images. The specific aspect of MAP approach, in formula, against MLEM is brought by the existence of a prior probability of reconstructed images through Bayes theorem. Namely, images are reconstructed via an iterative process to maximize the *a posteriori* probability function *P*(***μ*** ^∗^|***y***):

1 $$\begin{array}{*{20}l} P(\boldsymbol{\mu}^{*} | \boldsymbol{y}) = \frac{P(\boldsymbol{y} | \boldsymbol{\mu}^{*})P(\boldsymbol{\mu}^{*})}{P(\boldsymbol{y})}, \end{array} $$

where *P*(***y***|***μ*** ^∗^) is the probability of observing the projection data set ***y*** at the given expectation of the image ***μ*** ^∗^ having the prior probability *P*(***μ*** ^∗^). The observed projection *y*_*i*_ in a detector element at a certain projection angle is related to the corresponding photon count *n*_*i*_ as follows:

2 $$\begin{array}{*{20}l} n_{i} = n_{0} {\mathrm e}^{-y_{i}}, \hspace{1em}i = 1,2, \cdots, M. \end{array} $$

Moreover, the expected value is expressed as

3 $$\begin{array}{*{20}l}  n^{*}_{i} = n_{0} {\mathrm e}^{-y^{*}_{i}}, \hspace{1em}i = 1,2, \cdots, M, \end{array} $$

where *M* is the total number of projection elements given by the product of the number of detector pixels and the number of projection angles, *n*_0_ is the constant number of photons generated by the X-ray source, and *i* means *N*_*element*_×(*θ*−1)+*c*, where *N*_*element*_ is a total number of detector elements, *θ* is a projection number, and *c* is the specific detector element number. Assuming a monochromatic spectrum for the X-ray beam, the relationship between the image and the projection becomes linear:

4 $$\begin{array}{*{20}l}  \boldsymbol{y}^{*} = \boldsymbol{A}\boldsymbol{\mu}^{*}, \end{array} $$

where ***y*** ^∗^ represents the expected projection set, and ***A*** is the system matrix that consists of the voxel pass lengths corresponding to ***μ*** ^∗^. With Eq. (3), ***μ*** ^∗^ corresponds to the distribution of the attenuation coefficients in the assumed monochromatic X-ray energy spectrum. We also assume that the number of photons measured at the detector obeys a Poisson distribution:

5 $$\begin{array}{*{20}l} p(n_{i}) = \frac{(n^{*}_{i})^{n_{i}}}{n_{i} !} {\mathrm e}^{-n^{*}_{i}}. \end{array} $$

Applying Eq. (3), the probability *P*(***y***|***μ*** ^∗^) can be written as

6 $$\begin{array}{*{20}l} P(\boldsymbol{y}|\boldsymbol{\mu}^{*}) = \prod\limits_{i=1}^{M} \frac {(n_{0} {\mathrm e}^{-y_{i}^{*}})^{n_{i}}} {n_{i} !} {\mathrm e}^{-n_{0} {\mathrm e}^{-y_{i}^{*}}}. \end{array} $$

Now the *a posteriori* probability function Eq. (1) is described as follows:

7 $$\begin{array}{*{20}l}  P(\boldsymbol{\mu}^{*}|\boldsymbol{y}) = \left(\prod\limits_{i=1}^{M} \frac {(n_{0} {\mathrm e}^{-y_{i}^{*}})^{n_{i}}} {n_{i} !} {\mathrm e}^{-n_{0} {\mathrm e}^{-y_{i}^{*}}} \right) P(\boldsymbol{\mu}^{*}) /{P(\boldsymbol{y})}, \end{array} $$

and the logarithm of Eq. (7) is

8 $$ \begin{aligned} \ln{P(\boldsymbol{\mu}^{*}|\boldsymbol{y})}&= \sum\limits_{i=1}^{M} \left(n_{i} \ln{n_{i}} - {n_{i}}{y_{i}^{*}} -{n_{i}^{*}} -\ln(n_{i} !) \right)\\ &\quad +\ln{P(\boldsymbol{\mu}^{*})} - \ln{P(\boldsymbol{y})}. \end{aligned}  $$

Then, the maximization process of the logarithm of *P*(***μ*** ^∗^|***y***),

9 $$\begin{array}{*{20}l} \boldsymbol{\mu}^{*} & = \underset{\boldsymbol{\mu}^{*}}{\text{arg~max}} \left[ \ln{P(\boldsymbol{\mu}^{*}|\boldsymbol{y})} \right] \hspace{2mm}\text{subject\hspace{1mm}to} \hspace{2mm} \boldsymbol{\mu}^{*} > \boldsymbol{0}, \end{array} $$

gives reconstructed images. Hereafter the term ln*P*(***μ*** ^***∗***^) in Eq. (8) is regarded as the regularization or constraint term, *λ* *R*(***μ*** ^***∗***^), discussed in the next section. *λ* is the regularization parameter that describes the trade off between the regularization function term and the data fidelity term.

### Brief review of the constraint term used in total-variation-based compressed sensing (TVCS) and prior-image constrained compressed sensing (PICCS)

The meaning of *R*(***μ***) was originally the probability *P* of the volumetric image ***μ***, but it can actually be considered as the regularization term for image reconstruction. In the context of both TVCS and PICCS, the *ℓ*_1_-norm of a TV-operated image has been widely utilized: [23, 24]

10 $$ {\begin{aligned} &\|\Psi\boldsymbol{\mu} \|_{1}\\ &= \sum\limits_{x,y,z} \sqrt{(\mu_{x,y,z}\,-\,\mu_{x+1,y,z})^{2}\,+\,(\mu_{x,y,z}-\mu_{x,y+1,z})^{2} +(\mu_{x,y,z}-\mu_{x,y,z+1})^{2} + \epsilon}, \end{aligned}}  $$

where *ε* is a small constant that ensures that the total variation is differentiable at the origin. The TV operator *Ψ* is known as a sparsifying transform, and its *ℓ*_1_-norm is to be minimized in compressed sensing.

The difference in computation between TVCS and PICCS appears to be in the choice of the regularization term. If one chooses the reconstructed image itself as ***μ***, it should be the TV term. In contrast, if one chooses the difference between the reconstructed and prior images, ***μ***−***μ***_*prior*_, it is regarded as the prior-image constraint (PIC) term. Including both, *R*(***μ***) can be expressed as

11 $$\begin{array}{*{20}l} R(\boldsymbol{\mu})=\alpha\|\Psi\boldsymbol{\mu} \|_{1} + \beta\|\Psi(\boldsymbol{\mu}-\boldsymbol{\mu}_{prior})\|_{1}, \end{array} $$

where *α* and *β* are the weights of the TV and PIC terms, respectively. In the case of image reconstruction using PICCS, both constraint terms are combined and used as a weight; the values of 0.09 and 0.91 were respectively proposed for the TV and PIC terms by Chen et al. [23] TVCS reconstruction is implemented with the values of 1.0 and 0.0 for *α* and *β*, respectively.

### Time-ordered chain graph model (TCGM)

**Fig. 2:**
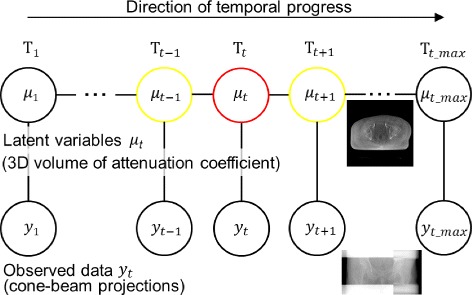
Concept of the time-ordered chain graph model. In the figure, ***μ***_*t*_ represents the image volume of the *t*-th time phase, and ***y***_*t*_ represents the *t*-th group of cone-beam projections. The sample images of them are also shown in the right side of the figure

In this study, we consider non-periodic time-ordered phenomena such as gastrointestinal peristaltic motion and the time progression of a contrast agent. Image reconstruction is implemented by dividing several time phases, and the time-ordered volumetric images can be obtained. In this context, it is likely that the time-adjacent volumetric images do not differ from each other very much. Thus, it would be justifiable to include the correlation between the time-adjacent volumetric images as the prior information in the reconstruction scheme. In the manner of the graphical model, the lowest-order correlation is expressed using the undirected graphical model shown in Fig. 2. In the figure, ***μ***_*t*_ represents the image volume of the *t*-th time phase, and ***y***_*t*_ represents the *t*-th group of cone-beam projections described by ***A***.

As shown in Fig. 2, ***μ***_*t*_ and ***y***_*t*_ are the latent variables and observed data, respectively. Assuming the continuous measurement of projections, ***y***_*t*_ can be regarded as one projection angle datum for each *t*-th time phase and regarded as a group of several continuous projection angle data when assuming volumetric image reconstruction.

Figure 2 demonstrates the concept of the TCGM, in which the state of the *t*-th time phase located between the (*t*−1)-th and (*t*+1)-th time phases is constrained by the image objects of the previous time phase ***μ***_*t*−1_ and the next subsequent time phase ***μ***_*t*+1_. In all time phases, the images are renewed during the iteration step so that the constraints can be also dynamically changed. That is, the converged images of all time phases are concurrently obtained.

A term constrained from the TCGM can be included in *R*(***μ***) as follows:

12 $$\begin{array}{*{20}l} R(\boldsymbol{\mu})=\alpha\| \Psi\boldsymbol{\mu} \|_{1} + \beta\|\Psi(\boldsymbol{\mu}-\boldsymbol{\mu}_{prior})\|_{1} + \gamma\|\Delta\boldsymbol{\mu}^{T}\|_{1} \end{array} $$

where *Δ* ***μ*** ^*T*^ represents the TCGM regularization term, which can be represented as a TV form of a subtracted image as

13 $$\begin{array}{*{20}l} \Delta\boldsymbol{\mu}^{T} = \Psi(\boldsymbol{\mu}_{t} - \boldsymbol{\mu}_{t-1}) + \Psi(\boldsymbol{\mu}_{t} - \boldsymbol{\mu}_{t+1}). \end{array} $$

The first term on the right hand side of Eq. (13) is the distribution of the spatial difference between the previous and current phases, whereas the second term is that between the current and subsequent phases. In the case of the first and the last phases, the adjacent phase exists only in one side and *Δ* ***μ*** ^*T*^ is formed as doubled TV form of one-sided subtracted image.

The parameter set (*α*,*β*,*γ*) in Eq. (12) controls the relative weight of the three sparsity-promoting terms in the objective function. In this study, (*α*,*β*,*γ*) was chosen to be (1.0,0.0,0.0),(0.1,0.9,0.0), and (0.1,0.0,0.9), for TVCS, PICCS, and the TCGM, respectively. TVCS is used to create the initial images in PICCS and the TCGM. In addition, TVCS-reconstructed images are used as the prior-image constraint in PICCS.

### Projection data sets for image reconstruction

**Fig. 3:**
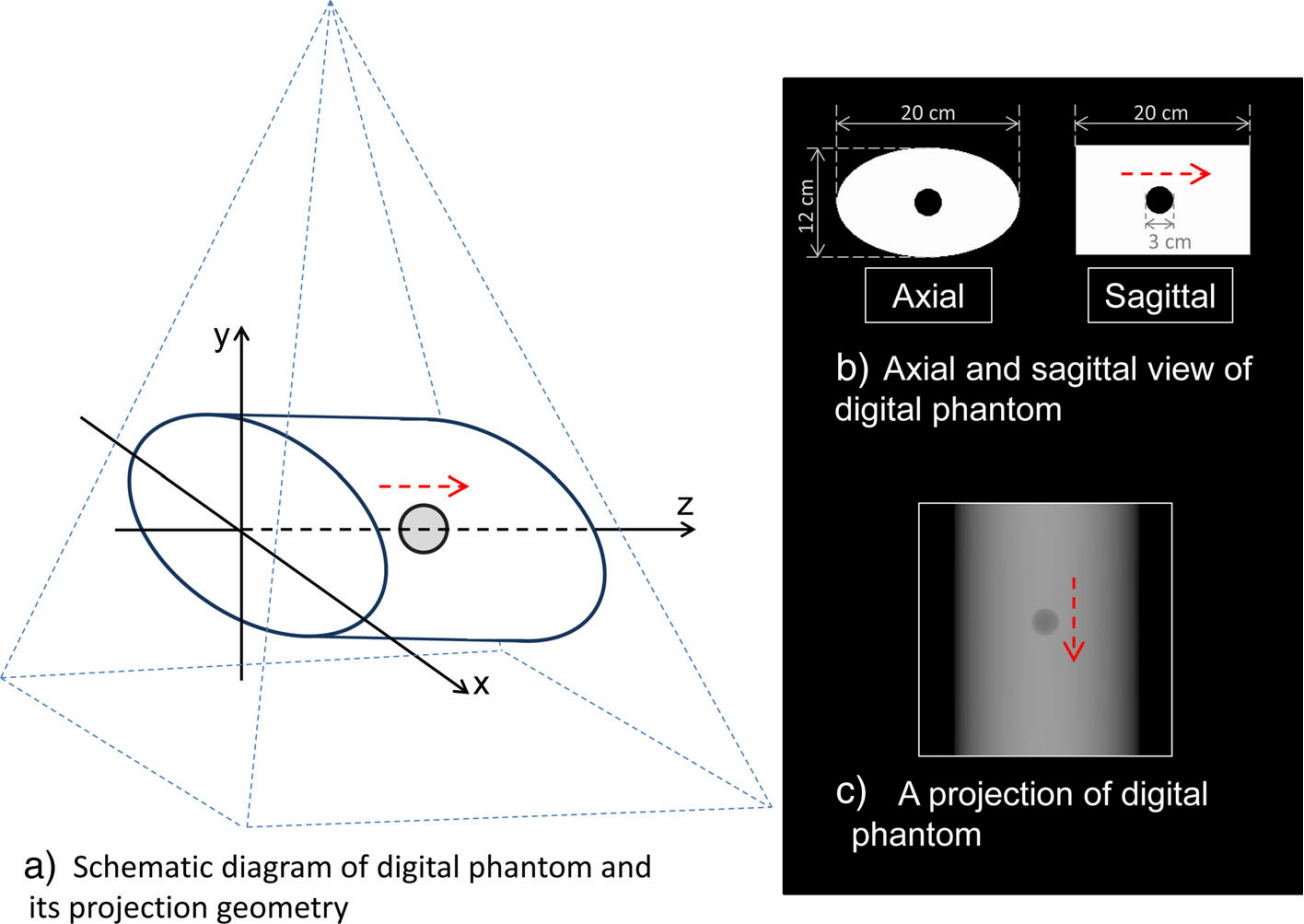
Schematic of the digital phantom and its projection geometry. **a** projection geometry and the phantom containing a moving sphere, **b** axial and sagittal views of the original digital phantom, and **c** one of the virtually created projections. An air sphere with a diameter of 3 cm was moving 3 cm along the longitudinal axis during one rotation of the projection source

Two kinds of projection data sets –a digital phantom and two clinical patients– were used for non-periodic time-ordered 4D image reconstruction. An elliptic cylinder containing a moving air sphere was used for the digital phantom, and cone-beam projection data for a 360° angular range with 512 ×512 elements were virtually created in one-degree intervals. The diameter of the air sphere was set as 3 cm, and the sphere moved by 3 cm along the longitudinal axis at a constant speed and without deformation, as shown in Fig. 3. Two kinds of the speed of the sphere were simulated: 3 cm-motion in 60 s and 3 cm-motion in 30 s. The former corresponds to 3 cm-motion in 360° rotation of the projection source, and the latter corresponds to 3 cm-motion during 180° rotation (from 90° position to 270° position of the projection source). Those speeds simulated 0.5 mm/s and 1.0 mm/s of the sphere motion, respectively. The projections were created as a sum of the product of voxel values and path lengths through the ray from the projection source to the center of each detector cell, and scattered x-rays and cell size of the detector were not taken into account.

Two patients’ projection data sets acquired for IGRT using the XVI system (version 4.2) mounted on the Synergy linac gantry were prepared. The data were acquired as pre-treatment CBCT imaging for the purpose of patient setup for prostate IMRT, and the chosen data sets contained the motion of rectal gas during projection acquisition. The acquisition time was approximately 120 s (696 projections) for patient A and 60 s (345 projections) for patient B.

The XVI system has three types of collimators, S, M, and L, which allow for the reconstruction of small, medium, and large fields of view (FOVs), respectively. With S collimator cassettes, the center of the kV radiation field is in line with the central axis of the kV X-ray source. With M and L collimator cassettes the kV radiation field is offset in the cross-line direction by 11.5 and 19 cm, respectively, at the flat-panel detector (FPD). The patients’ data sets in the present study were collected in the M FOV mode with an offset location of the FPD unit of 11.5 cm, whereas the geometry of the digital phantom projection assumed the XVI system with S FOV mode. The M FOV mode is frequently used for the projection acquisition of the abdominal or pelvic region, and the projections contain truncation [36–38]. Therefore, before the initial reconstruction using TVCS, the projection data were extended using the following procedure, which consists of three steps. First, the normal three-dimensional (3D) reconstruction containing the whole pelvis using all of the 360° projections was implemented. Second, virtual projections with 800 ×512 pixels were produced by the reprojection process of the reconstructed 3D image. Third, “extended" mosaic projections were created by combining real projections with “reproduced" peripheral area from virtual projections. Thus, the size of the extended 2D projection image was 800 ×512 for the clinical patient cases. This expansion allows for reconstruction of the whole body with 180° plus the fan angle, even in TVCS.

Image reconstruction was performed for nine continuous time phases with equal intervals in all TVCS, PICCS, and TCGM cases. An image matrix size of 256 ×256×60 with voxel size of 1 mm for the digital phantom reconstruction, and 400 ×400×60 with the same voxel size for patient data reconstruction was used. For TVCS, the projection data range was 180° + the fan angle = 200°; therefore, the projection data used in a certain phase were partly used in the reconstruction of the adjacent phases. For PICCS and the TCGM, the projection data range used was reduced by 90°. The projection data were still partly overlapped; however, the range was drastically decreased.

For the evaluation of our time-ordered reconstruction algorithms, the penumbral width of the moving air sphere along the CC direction was evaluated using the above digital phantom; reconstructed images of the digital phantom contained blurring of the air sphere due to longitudinal motion. The penumbral width of the longitudinal profile of the pixel values were compared among four reconstructions–normal 3D, TVCS (200° projection range), PICCS (90° projection range), and the TCGM (90° projection range). In this study, the penumbral width was defined as 10-90% penumbra of the pixel-inverse profile of the moving air cavity to compare the width of the time window.

**Fig. 4:**
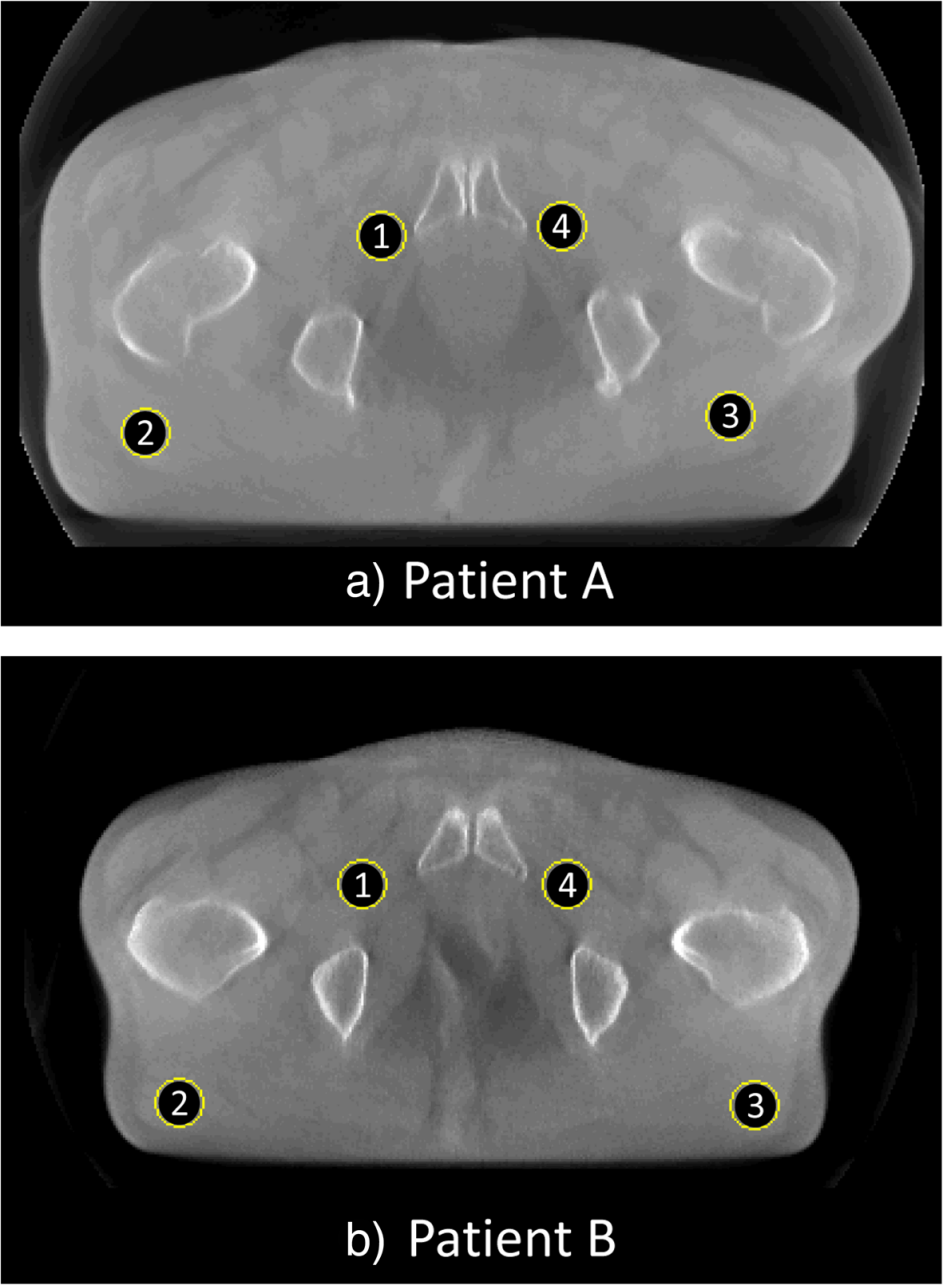
Location of the ROIs for pixel value consistency evaluation. Mean pixel values of ROIs 1 to 4 are evaluated for both patient A and B. Each circular ROI contains 316 pixels

Regarding reconstructed images of clinical patients, consistency of mean pixel values of TVCS (200°), PICCS (90°), and the TCGM (90°) were evaluated for both image series of patient A and B. The locations of four regions of interest (ROIs) are shown in Fig. 4. Those locations were chosen as the structure around each ROI was almost consistent. Each ROI contains 316 pixels, and root mean square errors (RMSEs) of mean pixel values inside the ROIs through nine phases were normalized by mean values of TVCS (360°) images.

## Results

### Results of digital-phantom image reconstruction

**Fig. 5:**
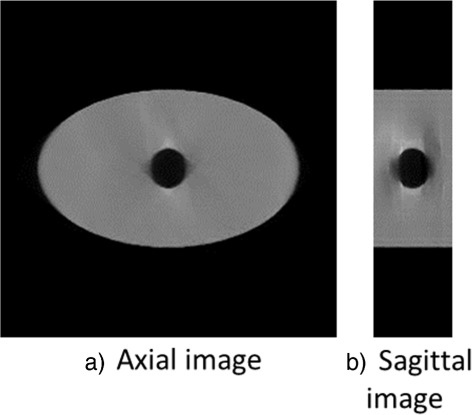
Reconstructed images of a digital phantom of the fifth phase out of nine phases in the case of the sphere speed of 3 cm/60 s, using the TCGM method. **a** reconstructed axial image and **b** sagittal image

The time-ordered images of the digital phantom were reconstructed using TVCS (200° projection range), PICCS (90° projection range), and the TCGM (90° projection range) as well as FDK (360° projection range). Figure 5 shows the reconstructed axial and sagittal images of the digital phantom with the sphere speed of 3 cm/60 s using the TCGM method (90° projection range). The pixel-value profiles of the reconstructed volume of the 5th time phase out of nine along longitudinal axis were measured, and their 10-90% penumbral widths are listed in Table 1. The three above methods provided much less blurring owing to motion compared with the normal 3D images reconstructed by FDK with full projection angles. In addition, PICCS and the TCGM provided less blurry images than TVCS with a 200° projection range mainly owing to the use of narrower projection images. In the case of the sphere speed of 3 cm/60 s, the results for PICCS and the TCGM were respectively 5.0% and 23.1% narrower than the image volume reconstructed with TVCS. The results of 3 cm/30 s reconstructed by PICCS and TCGM were 2.8% and 18.2% narrower than the result of TVCS, respectively. The TCGM yielded the smallest penumbral width, i.e., the best temporal resolution.

**Table 1:** Penumbral widths *W*_*penumbra*_ of the moving sphere along the longitudinal axis

Methods	*W*_*penumbra*_	
	3 cm/60 s	3 cm/30 s
FDK (360°)	22.0 mm	23.7 mm
TVCS (200°)	12.1 mm	21.4 mm
PICCS (90°)	11.5 mm	20.8 mm
TCGM (90°)	9.3 mm	17.5 mm

*W*_*penumbra*_ was defined as a width from 10 to 90% of the voxel value profile on the central axis, in the image of the fifth phase

### Results of patients’ pelvic image reconstruction

**Fig. 6:**
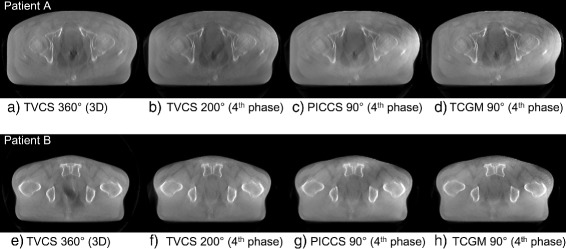
Reconstructed axial images of patient A and B with full FOV. Images from **a** to **d** are axial images of patient A reconstructed using TVCS (360°), TVCS (200°), PICCS (90°) and the TCGM (90°), respectively. Images from **e** to **h** are axial images of patient B reconstructed using the same reconstruction methods and ranges as patient A. Images **b** to **d** in patient A and **f** to **h** in patient B are the fourth phase out of nine. The display range is [0.019 0.022] mm ^−1^

**Fig. 7:**
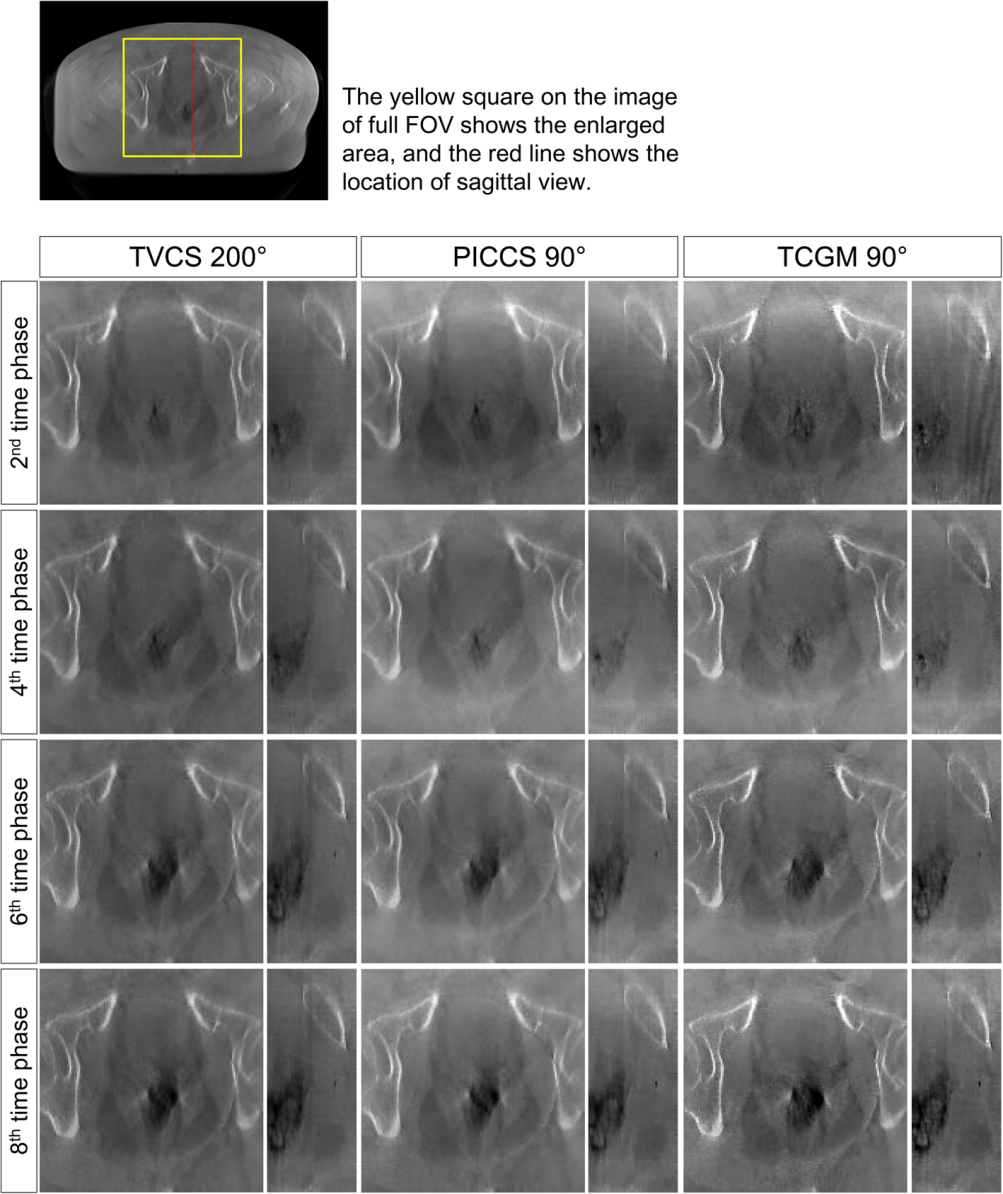
Enlarged image series of patient A. The reconstructed images of the 2nd, 4th, 6th, and 8th time phases using TVCS (200°), PICCS (90°) and the TCGM (90°) are shown in the first, second, and third columns, respectively. In each column, axial images are on the left and sagittal images are on the right. The yellow box and red line on the upper left image indicate the enlarged area of axial views and the location of sagittal views, respectively. The display range is [0.019 0.022] mm ^−1^

**Fig. 8:**
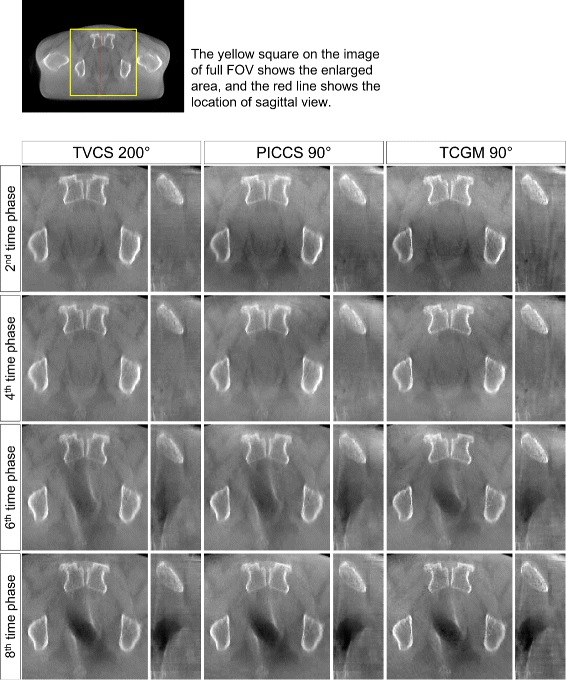
Enlarged image series of patient B. The reconstructed images of the 2nd, 4th, 6th, and 8th time phases using TVCS (200°), PICCS (90°) and the TCGM (90°) are shown in the first, second, and third columns, respectively. In each column, axial images are on the left and sagittal images are on the right. The yellow box and red line on the upper left image indicate the enlarged area of axial views and the location of sagittal views, respectively. The display range is [0.019 0.022] mm ^−1^

**Fig. 9:**
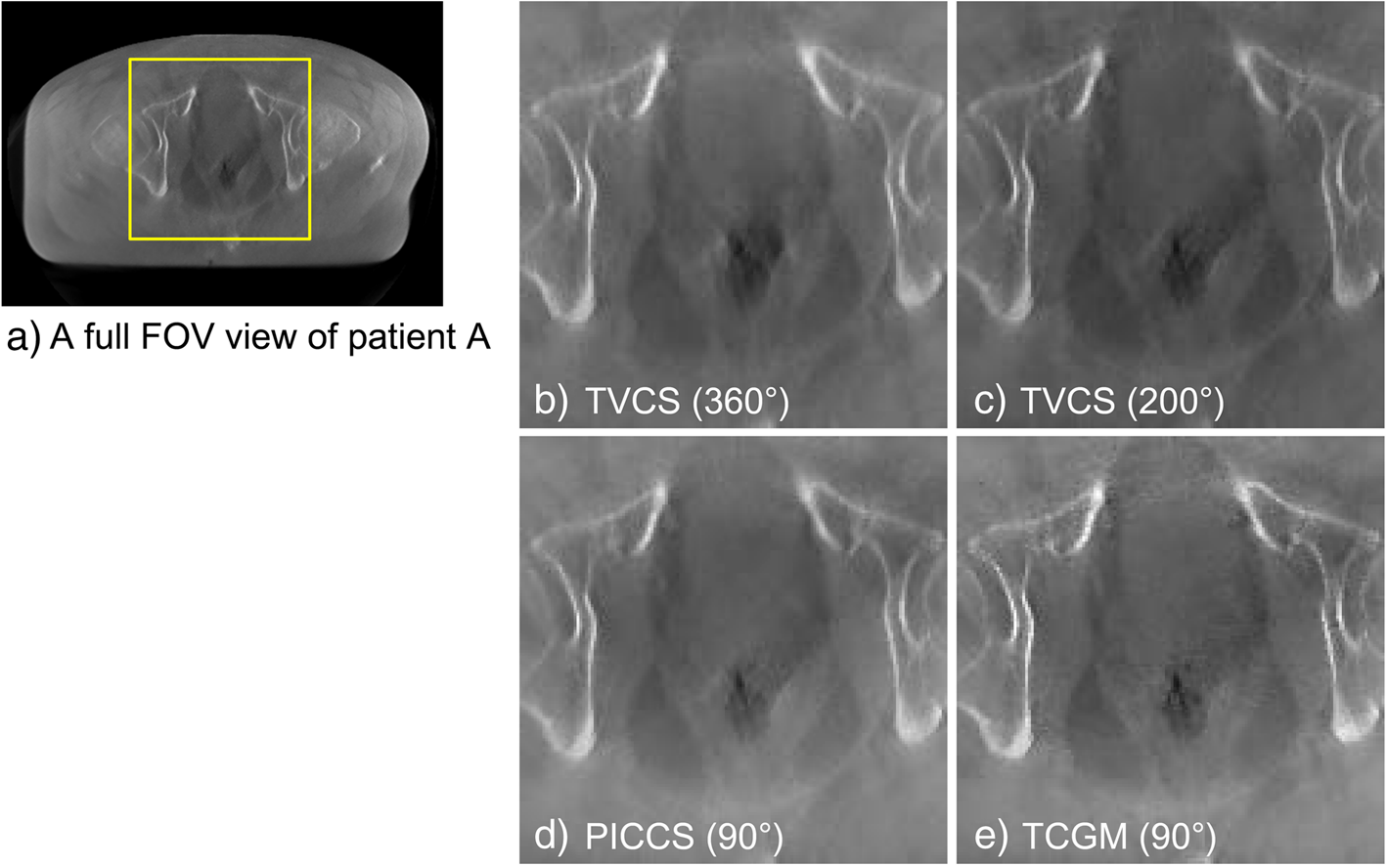
Comparison of motion artifact from rectum of patient A. The yellow box on image **a** shows the enlarged area of the axial images of patient A, the fourth phase. The images **b**, **c**, **d** and **e** are reconstructed using TVCS (360°), TVCS (200°), PICCS (90°) and the TCGM (90°). The display range is [0.019 0.022] mm ^−1^

Four-dimensional image reconstruction using TVCS (200° projection range), PICCS (90° projection range), and the TCGM (90° projection range) was applied to two clinical patients who underwent a CBCT scan in IGRT. The representative images are presented in Fig. 6 where only the 3D reconstructed image and the images of the fourth time phase are shown for both patient A and B. In both patient cases, the time-ordered images with full FOV are successfully reconstructed, though fluctuation of pixel values were observed. The evaluation of pixel value consistency regarding ROIs 1 to 4 in Fig. 4 were shown in Table 2. Relatively larger inconsistency was observed on the reconstructed images by PICCS and TCGM both using 90° range, compared to the TVCS (200°) reconstructed images. Especially in ROIs 2 and 3, which are located peripherally in the axial image, the fluctuation of pixel values reconstructed by PICCS and TCGM was relatively large, whereas the fluctuation of TVCS (200°) was quite small. The enlarged axial and sagittal views of patient A and B are shown in Figs. 7 and 8, respectively, where only the 2nd, 4th, 6th, and 8th time phases are shown. The motion of rectal gas could be observed for patient A (Fig. 7). Further, rectal gas appears in the images of the 6th and 8th time phase for patient B, but it is not visible in the 2nd and 4th time phase (Fig. 8). In particular, Fig. 9 demonstrate that the artifact from rectum is the weakest in the image (e) reconstructed by the TCGM, so the TCGM was the best method for reducing motion artifacts.

**Table 2:** Consistency evaluation of pixel values in 4D reconstructed images

Methods	Normalized RMSE							
	Patient A				Patient B			
	ROI1	ROI2	ROI3	ROI4	ROI1	ROI2	ROI3	ROI4
TVCS (200°)	3.5%	3.7%	3.5%	2.5%	5.2%	2.3%	0.7%	3.8%
PICCS (90°)	6.3%	7.8%	5.9%	6.9%	6.1%	6.8%	6.7%	8.9%
TCGM (90°)	7.0%	9.0%	6.4%	5.8%	7.5%	6.6%	7.8%	8.5%

Root mean square errors (RMSEs) of mean pixel values in each ROIs through nine phases, normalized by mean pixel values of TVCS (360°) images, are listed. The size and location of each ROI are shown in Fig. 4

**Fig. 10:**
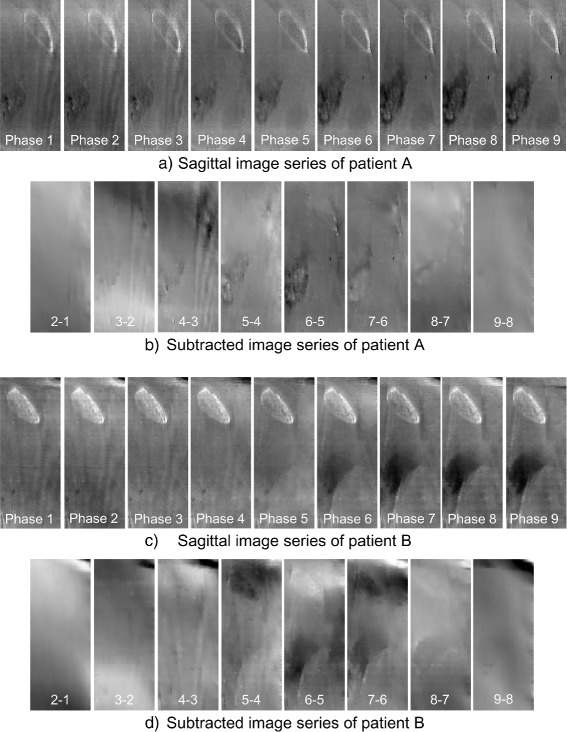
Sagittal image series and subtracted image series of patient A and B reconstructed using the TCGM (90°). The image series **a** and **c** are the sagittal image series of patient A and B through all phases, respectively. The image series **b** and **d** contain subtraction between adjacent phases of patient A and B, respectively

The time series of the enlarged sagittal views reconstructed using the TCGM (90°) and subtraction between adjacent phases are shown in Fig. 10. The sagittal views (a) and (c) indicate the transition of rectum through all time phases, and the subtraction between adjacent phases (b) and (d) indicates the timing of the significant change. These images demonstrate that the present approach can reconstruct patient situations before and after the sudden change.

## Discussion

All 4D CBCT techniques previously proposed for IGRT devices were based on the assumption that the motion of the object of interest was periodic, such as lung motion due to respiration. With this assumption, the motion signal (e.g., respiratory signal) was used to classify the projection images into several motion-phase bins so that the volumetric-image series were reconstructed using projection images within each bin. These reconstructed images admittedly included the dimension of time. However, they were still “averaged” 4D images over the gantry rotation and could not represent non-periodic organ motion, including the baseline shift in the lung-tumor location, which is obtained as time progresses.

To obtain the time-ordered images, the projection data should be classified by time-ordered phase bins. This idea would work even in the FDK reconstruction algorithm if a rapid scanning system was available.

In the IGRT device available in our institution, however, the gantry rotation speed was limited by 6.0°/s, though C-arm angiographic system has much faster options of the rotation speed [39, 40] and linac gantry speeds faster than 6.0°/s are currently possible under specific conditions according to IEC 60601-2-1. Therefore, the projection angle range in each time phase has to be as narrow as possible –much less than 180° + the fan angle– to ensure a high time resolution in the reconstructed images. The FDK algorithm could not work now, which requires a range of at least 180° + the fan angle, owing to the Shannon-Nyquist sampling theorem. A possible approach is to relax this requirement, and compressed sensing with prior information is a prime candidate. So far, an FDK reconstruction image with full projection data was used as the prior information. On the other hand, the time-ordered images should be mutually correlated. Therefore, it is natural to introduce the time-chain model as the prior information, as in the proposed method.

In this study, the advantage of the TCGM over TVCS and PICCS was confirmed by the results of the 10-90% penumbral width measurement in the digital phantom; the temporal window width becomes narrower in PICCS and TCGM, and TCGM was narrower than PICCS. The reason why the TVCS result is the widest is obvious; simply because resulted by the widest projection range of 200° was used in this study in comparison with those of PICCS and the TCGM, which were both 90°. It is noted that a range less than 200° (= 180° + the fan angle) cannot be applied in TVCS for successful image reconstruction of 40-cm FOV without any prior information, since projections of at least 180° range for all voxels in the FOV of 40 cm are needed [20]. Although the time-ordered images in the present study were reconstructed by extended projection image creation even in TVCS, TVCS has a disadvantage regarding the time resolution. On the other hand, the difference between PICCS and the TCGM is caused by the difference in the prior images used in the constraint terms. In the case of PICCS, an iteratively reconstructed image is always compared and constrained with the initial image, which is reconstructed using TVCS with a 200° projection range. In contrast, the TCGM uses the previous and subsequent images, which are being reconstructed in the same iterative process using 90° range of projections. Each 90° range overlaps with each other, which means that the related projection range is inherently narrower than that of PICCS.

The time resolution could be improved in both PICCS and the TCGM by increasing the number of time phases and narrowing the corresponding projection range, although there would be a trade-off regarding the image quality. We presented the nine-phase results with 90° projection ranges, and the results of clinical patients’ pelvic images were reasonably good; they showed rectal gas and flatus moving in the rectum. In the case of the TCGM, the images were reconstructed with fewer or weaker artifacts due to rectal gas, as shown in Fig. 9.

It is inherently possible to provide time-ordered 4D image series with a better temporal resolution with the presented TCGM method. The demonstrated results were reconstructed simply using just a 90° projection range and the weights of 0.1 and 0.9 for the TV and TCGM terms, and finding the optimized parameters is planned for future work. The behavior of the present method might be sensitive to the choice of weights. For the weight, one can introduce prior distributions and maximize probability function marginalized with respect to these hyper parameters to reconstruct images. More or less, this method definitely enables a reduction in the temporal window and provides image series of non-periodic time-ordered motion. The method should be used to understand the 4D dose distribution with in-treatment CBCT acquisition in adaptive radiotherapy scenarios [41–43].

The remarkable aspect of the present method is that the constraint term is based on the series of time-ordered statuses, which means that the *t*-th status is located between the (*t*+1)-th and (*t*−1)-th one and the constraint terms purely originate from the time-ordered change in the statuses. The present method can be improved by incorporating a deformation vector field to demonstrate organ motion and deformation using a more essential expression of motion.

It might be a limitation to reconstruct image volumes accompanied by sudden change and relatively fast motions, such as respiration, cough and swallowing, as the result of digital phantom study of double speed motion indicates. An initial motivation of the present method was to capture peristaltic activity of lower intestinal tract. So application of this method to the periodic motion like respiration might be resulting larger blurring compared to the 4D reconstruction from bunched projections using phase recognition introduced by the previous studies, [14, 15, 41] though base-line shift of diaphragm will probably be detected by the present method. Regarding sudden changes like cough and swallowing, it may be difficult to capture the time series of the motion using the present method. However the situation before and after the sudden change, e.g. status before and after cough or swallowing, can be reconstructed without strong motion artifacts caused by those sudden changes. Those images will probably have enough image quality in order for irradiated 4D dose reconstruction and evaluation of tumor shrinkage. Another limitation of the present method might be inconsistency of pixel values through all time phases. Pixel values, especially in peripheral areas of reconstructed FOV, might be fluctuated regarding projection ranges used in the reconstruction of particular phase. In the previous studies the methods to avoid those inconsistencies in dose reconstruction on CBCT images were already proposed, such as ROI mapping approach and WAB method [44]. Those method can be applied to the dose reconstruction with image sets reconstructed using the present method.

## Conclusion

A new concept of 4D CBCT reconstruction called the TCGM has been proposed, and reconstructed images obtained with this method have been compared to those obtained by TVCS and PICCS. The digital-phantom results demonstrated that the proposed method can provide 4D image series with a better temporal resolution compared to the two other methods. The clinical patients’ results show that the present method is applicable to the visualization of the motion of rectal gas and flatus in the rectum.
